# Retraction: Down-regulation of lncRNA XIST inhibits cell proliferation via regulating miR-744/RING1 axis in non-small cell lung cancer

**DOI:** 10.1042/CS20190519_RET

**Published:** 2025-01-30

**Keywords:** migration, miR-744, non-small cell lung cancer (NSCLC), proliferation, RING1, XIST

This article “Down-regulation of lncRNA XIST inhibits cell proliferation via regulating miR-744/RING1 axis in non-small cell lung cancer” DOI: 10.1042/CS-2019-0519 is being retracted from *Clinical Science* at the request of the Editor-in-Chief and the Editorial Board. This follows receipt of a notification from readers, alerting the Editorial Board to:

- Figure 2F A549 si-XIST-1 parts of which seem to appear in Figure 2F A549 si-XIST-2 panel with rotation and flipping transformation
- The images in Figure 2F (excluding A549 si-XIST-2) which seem to appear in Figure 4h from Liang et al, 2018 (DOI: 10.1038/s41419-018-0582-1)
- Figure 2G H23 pcDNA3.1-Vector panel part of which seems to appear in Figure 2G H522 pcDNA3.1-XIST panel
- Figure 4G H522 XIST-Mut cDNA + miR-744 mimins panel, parts of which seem to appear in Figure 4G H522 Vector cDNA + miR-744 mimics panel with rotation
- Figure 4G H23 XIST-Mut cDNA+miR-744 mimins panel parts of which seem to appear in H23 Vector cDNA+miR-744 mimics panel with flipping transformation
- Areas that appear to be duplicated within Figure 4G H522 XIST-Mut cDNA+miR-744 mimins panel, and separately within Figure 4G H522 Vector cDNA +miR-744 mimics panel

The Editorial Office has additional concerns for Figure 4G XIST-Mut cDNA+Control mimins panel which seems to appear as Figure 5d SW620 miR-NC panel from Shi et al, 2019 (DOI: 10.1038/s41419-019-1332-8) with rotation, and signs of digital editing in Figure 6E H522 β-catenin, c-myc, and cyclin D1 Western blots.

The authors have been contacted with regards to the retraction and have not responded to the journal’s queries or the concerns raised. Given the extent of the issues raised, the Editorial Board stand by the decision to retract the article.

